# Real-Time Live-Cell Imaging Technology Enables High-Throughput Screening to Verify in Vitro Biocompatibility of 3D Printed Materials

**DOI:** 10.3390/ma12132125

**Published:** 2019-07-02

**Authors:** Ina G. Siller, Anton Enders, Tobias Steinwedel, Niklas-Maximilian Epping, Marline Kirsch, Antonina Lavrentieva, Thomas Scheper, Janina Bahnemann

**Affiliations:** Institute of Technical Chemistry, Leibniz University Hannover, Callinstraße 5, 30167 Hannover, Germany

**Keywords:** real-time live-cell imaging technology, in vitro study, biocompatibility, 3D printing, flow cytometry, adipogenic mesenchymal stem cells

## Abstract

With growing advances in three-dimensional (3D) printing technology, the availability and diversity of printing materials has rapidly increased over the last years. 3D printing has quickly become a useful tool for biomedical and various laboratory applications, offering a tremendous potential for efficiently fabricating complex devices in a short period of time. However, there still remains a lack of information regarding the impact of printing materials and post-processing techniques on cell behavior. This study introduces real-time live-cell imaging technology as a fast, user-friendly, and high-throughput screening strategy to verify the in vitro biocompatibility of 3D printed materials. Polyacrylate-based photopolymer material was printed using high-resolution 3D printing techniques, post-processed using three different procedures, and then analyzed with respect to its effects on cell viability, apoptosis, and necrosis of adipogenic mesenchymal stem cells (MSCs). When using ethanol for the post-processing procedure and disinfection, no significant effects on MSCs could be detected. For the analyses a novel image-based live-cell analysis system was compared against a biochemical-based standard plate reader assay and traditional flow cytometry. This comparison illustrates the superiority of using image-based detection of in vitro biocompatibility with respect to analysis time, usability, and scientific outcome.

## 1. Introduction

3D printing has become a highly attractive tool with numerous different applications in the last decade. Already established technologies in the realm of rapid prototyping, 3D printing techniques are now increasingly being used to fabricate individually-designed devices in a comparatively easy, time and cost-effective way. Several 3D printing technologies are now available on the market, diverging mainly in the printing process and/or the physical state of the material bases utilized. The most established of these technologies create devices by melting and extruding thermoplastic filaments, fusing small particles of polymer powder together, or curing liquid resins via photopolymerization [1]. There are some fundamental similarities, however, for example, all 3D printing techniques make use of a “layer by layer” fabrication process, which facilitates almost unlimited complexity with respect to the final printed product.

Facilitated by 3D printing, the rise of rapid prototyping has great potential to accelerate the progression of biomedicine, biotechnology, and tissue engineering. Put differently, 3D printing permits the rapid fabrication of customized medical products and equipment, which can enable more individualized medical application [1,2,3]. The generation of implantable, highly porous 3D scaffolds has become an increasingly important concept within the field of tissue engineering [3,4]. Such porous, personalized scaffolds provide a suitable surface for patient-specific cells to proliferate under ideal conditions. Nevertheless, despite these recent advances in medical applications involving 3D printing, the in vivo use of 3D printed materials should still be treated with some degree of caution, given the tremendous complexity of interactions within the human body. One major challenge associated with introducing foreign material into an organic system is the concept of “biological compatibility” or “biocompatibility” [1]. A generally accepted definition of this concept was given by D. F. Williams in 1987, “Biocompatibility is the ability of a material to perform with an appropriate host response in a specific application” [5].

An “appropriate host response” includes a normal healing process, resistances to bacterial colonization or biofilm formation and the prevention of blood clotting [6]. The biocompatibility of all materials being considered for use in real-world biomedical applications must first therefore be carefully assessed and confirmed via in vivo and in vitro studies [6,7]. If leachables or extractables show a negative impact on mammalian cells in vitro, then a material cannot be characterized as biologically compatible [8]. International standards—such as ISO 10993—provide extensive information, which can be used to develop appropriate assays and otherwise inform about biocompatibility testing methods [9].

A variety of cell culture-based in vitro assays are available for investigation of cytotoxicity of materials. These methods vary widely, from analysis and counting of viable/dead cells via microscope to biochemical-based assays and flow cytometric analyses. Microscopic observations of changes in cell morphology and counting of viable/dead cells form the basis [7]. As vital dyes such as Trypan blue can only enter—and thereby mark out—cells with disrupted cell membranes, use of these dyes in tissue cultures allows researchers to visually distinguish living and dead cells [10]. Biochemical-based assays provide a more reliable and specific outcome [7,11]. Numerous commercial assays are available, each one dealing with a different process of cellular metabolism [7,12]. For example, the CellTiter-Blue^®^ assay (CTB assay) used within this study relies on the conversion of resazurin to the fluorescent product resorufin, which highlights the intracellular reduction potential of living cells [13]. However, although assays like this are widely used, they can only highlight the fundamental distinction between living and dead cells—they do not allow any further nuanced analysis into the different mechanisms by which cell death may occur [7]. Analyses of apoptosis and necrosis provide more detailed information on this front. The apoptotic pathway describes the mechanism of an internally “programmed” cell death [14,15]. By contrast, necrosis is a cellular death mechanism that has been triggered by external factors, such as injury or infection [14]. Both of these pathways display distinct morphological and biochemical features that can be observed and analyzed using specific fluorescence detection markers.

Due to the versatility of 3D printing technologies, a wide variety of printing materials—as well as post-processing procedures and surface finishing steps—are now being utilized. The materials can differ (for example) in their physical state, melting temperature, strength, and/or durability [1]. And the potential fields of application for any given method—as well as associated necessary post-processing or sterilization steps—are ultimately dependent on the properties of the underlying materials [7,16]. For example, materials with a high heat distortion temperature can be sterilized by thermal sterilization (autoclaving) for subsequent use in biological applications, while materials with a lower heat distortion tolerance require alternative sterilization procedures. Support materials such as wax are used by many 3D printing technologies to provide a scaffold with which to stabilize the building material. Following the printing process, this support material must be removed. The post-processing and removal of support material residues is also material-dependent, and can cause difficulties—especially with respect to detailed 3D structures and small channels (for example in microfluidic systems [17]. Depending on the post-processing and sterilization procedure, different end products with different properties can be obtained from the same material formulation. For applications in cell culture, every material formulation and post-processed product needs an individual investigation for biocompatibility. Accordingly, there is an apparent need for high-throughput screening assays.

This study seeks to help to fill in that gap by introducing real-time live-cell imaging technology as a fast, cost-effective and easy to use screening method to examine the in vitro biocompatibility of materials. To that end, a translucent clear, solid polyacrylate was printed via a high-resolution MultiJet 3D printing process, and was then post-processed to remove the supporting material. Following this post-processing procedure, three different disinfection and sterilization methods were examined, using ultra violet (UV) light as a physical sterilization method, as well as ethanol (70%, *v*/*v*) and sodium hypochlorite (2%, *v*/*v*) as chemical reagents. Afterwards, all of the post-processed objects were analyzed and screened for their suitability in cell culture applications by comparison of different in vitro biocompatibility methods. For biocompatibility evaluation, extraction media were obtained in accordance to ISO 10993:2012 standards and its impact on adipogenic mesenchymal stem cells (MSCs) was observed. Metabolic activity (representing cell viability) was assessed using a CellTiter-Blue^®^ (CTB) cell viability assay. Analyses of apoptotic and necrotic responses as a measure of biocompatibility were also performed in a comparative study, using both modern image-based live-cell analysis technology and traditional flow cytometry.

## 2. Materials and Methods

### 2.1. Experimental Procedure

**Figure 1:**
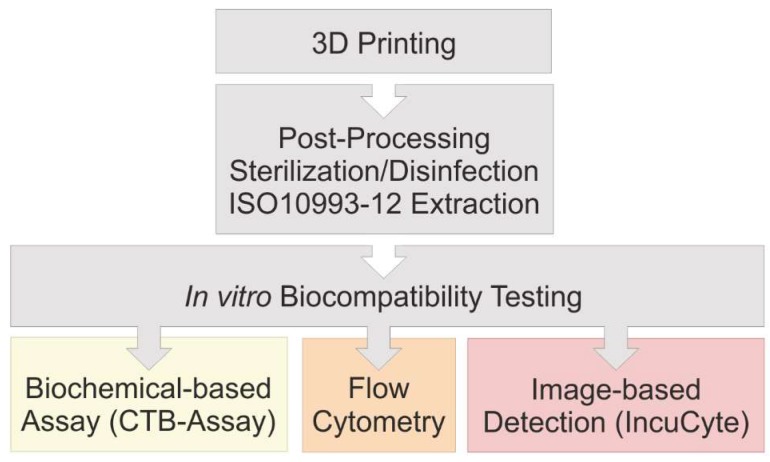
Flow chart of performed experiments. The in vitro biocompatibility of 3D printed material was evaluated using three different approaches.

After 3D printing of a translucent polyacrylate material was completed, the printed parts are then cleaned in post-processing steps and sterilized or disinfected, respectively, using three different approaches. According to ISO 10993:12, extraction media are obtained for studying the influence of the 3D printed material on MSCs in in vitro biocompatibility assays. Three different methods to assess in vitro biocompatibility were compared in this study: (1) A biochemical-based viability assay (CTB assay) in a standard plate reader; (2) traditional flow cytometry; and (3) novel image-based live-cell analysis. A schematic overview of the experimental procedure is shown in Figure 1.

### 2.2. 3D Printing

3D printed constructs were manufactured using the high-resolution MultiJet 3D printer ProJet^®^ MJP 2500 Plus (3D Systems, Rock Hill, SC, USA). The 3D printing material analyzed in this study is VisiJet^®^ M2R-CL (3D Systems, Rock Hill, SC, USA). It appears as a translucent clear, solid polyacrylate following a UV-curing process, and it is printed with a resolution of 800 × 900 dots per inch and a layer resolution of 32 µm [18,19]. As support material for the printing process, VisiJet^®^ M2-SUP is used. For studying the success of the post-processing and the potential influence of leachables, 5 × 5 × 5 mm cubes were printed—representing a total surface area of 1.5 cm^2^·ml^−1^. The known hazardous components in the liquid state of the present acrylic photopolymer material are 3-hydroxy-2,2-dimethylpropyl, 3-hydroxy-2,2-dimethylpropionate, the polymerization initiator diphenyl(2,4,6-trimethylbenzoyl) phosphine oxide, and monofunctional urethane acrylate. Together with the constituent tricyclodecane dimethanol diacrylate, these components are all listed as being potentially harmful to aquatic organisms and/or as otherwise potentially causing adverse effects on aquatic environments in their liquid state (i.e., before polymerization) [19]. During the printing process, the polyacrylic material is polymerized by UV light—after which it can be declare harmless. No additional information regarding the material was provided by the manufacturer.

### 2.3. Post-Processing of 3D Printed Objects

**Figure 2:**
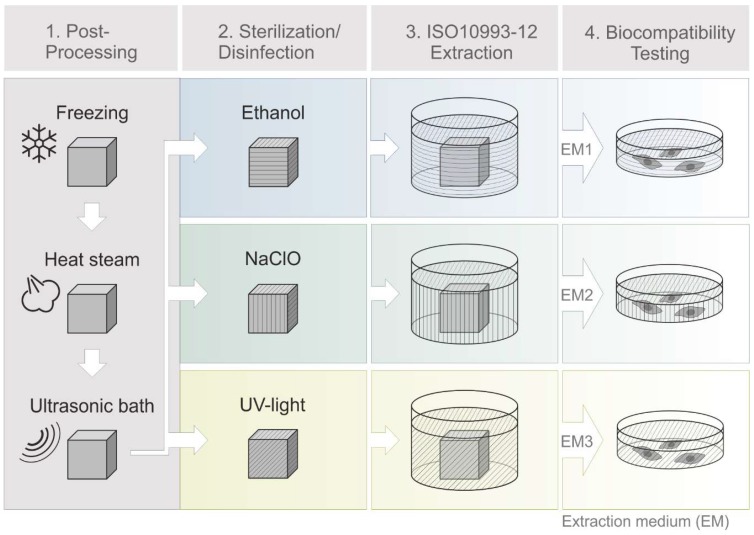
Schematic process of 3D printing, post-processing and extraction medium preparation. Cleaning steps (**1**): freezing of 3D printed objects (15 min, −18 °C), heat steam in a water bath (45 min, 65 °C), ultrasonic bath with detergent (30 min, 65 °C). Sterilization steps (**2**): disinfection in ethanol (70%, *v*/*v*, 1 h, RT) or sodium hypochlorite (2%, *v*/*v*, 1 h, RT) or UV light exposure (1 h, RT). Biocompatibility testing steps (**4**) then followed an incubation of 3D printed objects in cell culture medium according to EN ISO 10993-12 (2012) (**3**). (EM = extraction medium). EM 1: EM obtained by incubation of 3D printed material treated with ethanol (70%, *v*/*v*) in a disinfection process. EM 2: EM obtained by incubation of 3D printed material treated with sodium hypochlorite (2%, *v*/*v*) and EM 3: EM obtained by incubation of 3D printed material sterilized by UV light.

All steps of the post-processing are shown in Figure 2, and they include freezing the printing plate for 15 min at −18 °C, and placing 3D printed objects in a heat steam bath of the EasyClean unit (3D systems, Rock Hill, SC, USA) for 45 min at 65 °C and incubation in an ultrasonic bath (Bandelin electronic, Berlin, Germany) with detergent (Fairy Ultra Plus, Procter and Gamble, CT, USA) for 30 min at 65 °C. Deionized water, provided by Arium^®^ (Sartorius Stedim Biotech GmbH, Göttingen, Germany), was used in all experiments.

### 2.4. Sterilization/Disinfection of 3D Printed Objects

One disadvantage of many 3D printed materials is their relatively low heat distortion temperature and their corresponding incompatibility with thermal sterilization approaches [19,20,21]. However, a guaranteed sterile and disinfected product is necessary for the use in biomedical applications [22,23]. The polyacrylic material used in this study has a heat distortion temperature around 80 °C, as a result the most common sterilization method (autoclaving) is not a possibility [19,24,25]. But physical and chemical procedures can also be used to sterilize and disinfect materials [24]. In this study, two different methods for chemical disinfection were used: The product was subjected to incubation in either ethanol (Carl Roth GmbH und Co. KG, Karlsruhe, Germany), 70%, *v*/*v*, or sodium hypochlorite, 2%, *v*/*v*, (Carl Roth GmbH und Co. KG, Karlsruhe, Germany), for 1 h at room temperature. In addition, UV irradiation (UV Sterilization Cabinet KT-09DC, Alexnld, Tiberias, Israel, 6 W, λ = 266 nm) was also used as a physical sterilization method. In order to cover every side of the 3D printed cubes with UV light, the cubes are turned around within a total of 1 h of UV light exposure at room temperature. After sterilization or disinfection procedure all cubes were washed thoroughly with sterile phosphate-buffered saline (PBS).

### 2.5. Preparation of Extraction Media (EM) for Biocompatibility Studies

Potential leaching properties of the 3D printed material, or remaining support material, were evaluated by obtaining extraction medium (EM) according to EN ISO 10993-12:2012 (Biological evaluation of medical devices—art 12: Sample preparation and reference materials). After post-processing, the aforementioned 3D printed cubes were incubated in cell culture medium Minimum Essential Medium Eagle, with alpha modification (α-MEM) (Thermo Fisher Scientific Inc., Waltham, MA, USA) containing 10% human serum (c.c.pro GmbH, Oberdorla, Germany) and 1% Gentamicin (PAA Laboratories GmbH, Pasching, Austria), for 72 h at 37 °C with a surface area/volume ratio of 3 cm^2^·ml^−1^. The obtained medium is referred to as extraction medium (EM). EM obtained by incubation of 3D printed material treated with ethanol (70%, *v*/*v*) in post-processing process is referred to as “EM 1.” EM obtained by incubation of 3D printed material treated with sodium hypochlorite (2%, *v*/*v*) is hereafter referred to as “EM 2”, and EM obtained by incubation of 3D printed material sterilized by UV light is referred to as “EM 3.” Cell culture medium incubated for 72 h at 37 °C without 3D printed objects served as a control for all biocompatibility experiments.

### 2.6. Cell Line and Cell Culture Conditions

For all experiments, human adipogenic tissue-derived mesenchymal stem cells (MSCs) were used. After obtaining the donor’s informed written consent, as approved by the Institutional Review Board (Hannover Medical School) with the reference number 3475-2017, adipose tissue was received following abdominoplasty surgery. After isolation, MSCs have been extensively characterized by surface marker analysis and functional properties as described earlier [26]. Cultivation of MSCs was performed in cell culture medium in a 5% CO_2_, 21% O_2_, humidified atmosphere at 37 °C (Heracell 150i incubator, Thermo Fisher Scientific Inc., Waltham, USA). The MSCs were routinely maintained in 75 cm^2^ cell culture flasks (Corning, CellBind Surface, Corning, NY, USA), and then harvested at about 85% confluency by accutase treatment (Merck KGaA, Darmstadt, Germany) for detachment [26]. 24 h prior to the start of an experiment, cells were seeded in 6-, and 96-well plates (at a density of 18,000 cells·cm^−2^ and 1100 cells·cm^−2^, respectively) (Sarstedt AG and Co. KG, Nürnbrecht, Germany). Experiments were performed with cells of passages two to six.

### 2.7. CellTiter Blue^®^ (CTB) Viability Assay in Fluorescence Plate Reader

For indirect evaluation of cell viability using a standard method, a CellTiter-Blue^®^ cell viability assay (Promega, GmbH, Mannheim, Germany) was performed using the background and standard controls specified in the accompanying manual. Metabolically active cells are able to reduce blue resazurin into a purple, fluorescent resorufin via action of numerous redox enzymes in different intracellular compartments [13,27,28]. The fluorescence intensity produced by this reaction is therefore indicative of the number of viable cells. The product formation is monitored at an extinction wavelength of 544 nm, and an emission wavelength of 590 nm, using a fluorescence plate reader (Fluoroskan Acent, Thermo Fisher Scientific Inc., Waltham, MA, USA). MSCs were seeded in 96-well plates at a density of 8000 cells/well in 100 µl cell culture medium and incubated for 24 h at 37 °C in a humid atmosphere supplemented with 5% CO_2_. Subsequently, the MSCs were cultivated in the related extraction medium (see Section 2.5) or control medium for 24 h. After 24 h, extraction or control medium was removed, 100 µl fresh culture medium containing 10% CTB stock solution was added to each well and the MSCs were incubated for 1.5 h before measuring the fluorescence in a plate reader. Each experiment was repeated 13 times (*n* = 13).

### 2.8. Cell Viability Analysis by Flow Cytometry

Flow cytometry represents the traditional method used to monitor and quantitatively examine cell apoptosis and necrosis [29]. The BD FACSAria™ Fusion (Becton Dickinson, Franklin Lakes, NJ, USA) used in this study contains four lasers with numerous filters, which allow for a combination of multiple fluorescence markers within one sample. The basic principle of a flow cytometer is the analyses of hydrodynamically focused single cells that pass orthogonally through a bundled laser beam of a suitable wavelength. As they pass through the laser beam, the cells can be identified and classified by their physical characteristics (i.e., according to cell size, granularity, or specific fluorescence labeling) [30].

#### 2.8.1. Sample Preparation

MSCs were seeded at a density of 18,000 cells·cm^−2^ in 6-well plates and then incubated for 24 h at 37 °C under 5% CO_2_. Before related extraction media or control medium was used (as described below in Section 2.5), MSCs were washed once with PBS to remove non-adherent cells. MSCs were then cultivated in correspondent media for another 24 h. Cell samples for cell counting and flow cytometry experiments were obtained by detachment of adherent cells using accutase treatment. Before dyeing and analysis, the detached cells were sedimented by centrifugation for 5 min at 200× *g* and then resuspended in fresh culture medium [31,32]. The cell number and viability was estimated viw cell counting using a 0.4% Trypan blue stain (*n* = 4) in a haemocytometer (Brand GmbH + Co. KG, Wertheim, Germany) [10]. Trypan blue can be used to visually identify cells with disrupted cell membranes since dead or damaged cells possess a compromised membrane integrity which allows the dye to enter the cell and visibly mark it as distinct from a healthy living surrounding.

#### 2.8.2. Measurement and Quantification of Apoptosis and Necrosis

MSCs were centrifuged for 5 min at 200× *g*, resuspended, and then washed with PBS twice. Necrotic cells were marked and identified using the SYTOX^®^ AADvanced™ Dead Cell Stain, which is provided in the CellEvent™ Caspase-3/7 Green Flow Cytometry Assay Kit (Thermo Fisher Scientific Inc., Waltham, MA, USA). These cells were stained as instructed in the manual. Necrotic cells possess disrupted cell membranes which allow the Dead Cell Stain to enter the cell and intercalate in DNA structures, thereby visually marking out the cell. Necrosis can be measured at an excitation maximum of 546 nm and an emission maximum of 647 nm. Apoptotic cells express and activate the enzymes caspase-3 and caspase-7 [33]. Hence, apoptosis can be evaluated by the detection of active caspase-3/7 using the CellEvent™ Caspase-3/7 Green Stain, provided in the same assay kit. The corresponding green fluorescence signal has an excitation maximum of 511 nm and an emission maximum of 533 nm, and was captured with appropriate laser and filter settings using a BD FACSAria™ Fusion flow cytometer. The same number of cells were stained in each sample, in order to maintain an equal distribution of fluorescence reagents to cells. To represent a positive control for apoptosis, 50 µm cisplatin (cisplatin-induced apoptosis) was also added to the cells (control experiments were performed in triplicate). Cisplatin is a platin derivative that blocks DNA synthesis, induces apoptosis via p53-dependent and independent signaling mechanisms, and activates caspase-3. It is a well-known DNA-alkylating antitumor agent which is used as a chemotherapeutic drug [34,35]. MSCs cultivated in normal cell culture medium, without contact to 3D printing material, served as a negative control. The MSCs were cultivated for a period of 30 h, with cell samples taken every 4–6 h (*n* = 6). Cell samples were handled and counted via the Trypan blue exclusion method (described in Section 2.8.1). The BD FACS Diva™ Software v8.0 (Becton Dickinson, Franklin Lakes, NJ, USA) was used for analysis. Flow cytometry analysis is predicated on the principle of “gating”, by placing gates around cell populations with common characteristics, different cell populations can be segregated and selected for further investigation. Here, a uniform gating strategy was used for all experiments in order to separately analyze and quantify apoptotic, necrotic and living cells. Necrotic and apoptotic cells, respectively, possess higher red and green fluorescence signal intensities compared with living cells. Gates were determined based on both positive and negative cell controls. At least 10,000 events per sample were analyzed with an “event” being defined as a single particle detected by the system. The experiment was performed with three biological replicates.

### 2.9. Cell Viability Analysis by Real-Time Live-Cell Imaging System

**Figure 3:**
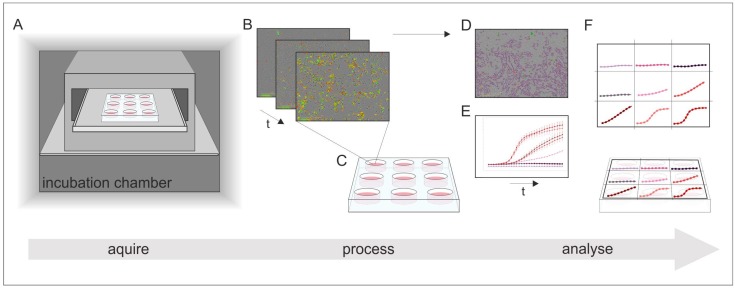
Schematic illustration of the working process of real-time live-cell analysis. (**A**) Placing of the real-time live-cell imaging system inside a cell culture incubator; (**B**) automatically acquire images over time; (**C**) receive images of all locations in the culture vessel at once; (**D**) imaging masks identify regions of interest; and (**E**) the results can be monitored in real-time and (**F**) display quantitative and kinetic analyses of all culture vessels at once.

The IncuCyte^®^ Live-Cell Analysis System (Sartorius Stedim Biotech GmbH, Göttingen, Germany) is an image-based real-time system that allows for an automatic acquisition and analysis of cell images. With the use of two lasers, both phase contrast as well as fluorescence images can be captured. The entire system is placed inside a cell culture incubator in order to guarantee controlled cultivation conditions during real-time monitoring. Phase contrast and fluorescence images are automatically recorded and analyzed using customized software tools in the IncuCyte^®^ S3 image analysis software (Sartorius Stedim Biotech GmbH, Göttingen, Germany). With pre-defined imaging masks, fluorescence signals of the recorded images are then analyzed and counted. Parameters such as minimum fluorescence signal intensity are considered and defined in advance (e.g., to exclude diffuse background noise from the evaluation). The same imaging masks are applied to all acquired images. The data is exported as Counts/Image, which represents the counted fluorescence signals with respect to a single image. The applied dynamic image processing and analysis enables quantitative real-time analyses of fluorescence signals in an imaging field. In addition, by using pre-defined cell-specific imaging masks containing information on cell size and shape, cell growth can be monitored in real-time, by analyzing the occupied area of an imaging field in phase contrast images. Accordingly, this system provides both quantitative and kinetic data. A schematic workflow of the real-time live-cell imaging system is shown in Figure 3.

#### 2.9.1. Sample Preparation

MSCs were seeded in 96-well plates at a density of 8000 cells/well in 100 µl cell culture medium and then incubated for 24 h at 37 °C in a humid atmosphere supplemented with 5% CO_2_. Staining reagents for quantification of apoptosis and necrosis were diluted in respective cell culture medium obtained as described in Section 2.5. Before the staining reagents containing media were added to these cultivation wells, the old medium was first discarded and all non-adherent cells were removed by a washing step with PBS.

#### 2.9.2. Measurement and Quantification of Apoptosis and Necrosis

A quantitative analysis of apoptosis and necrosis of MSCs over time was ascertained during the cultivation in extraction medium 1 (EM 1) and extraction medium 2 (EM 2), with regular cell culture medium serving as a control. Staining of the cells was performed using the IncuCyte^®^ Cytotoxicity and Apoptosis Detection Kits (Sartorius Stedim Biotech GmbH, Göttingen, Germany), according to the manufacturer’s protocols. Real time measurement of necrosis is based on the cell membrane integrity—i.e., the same principle as used for necrosis detection in flow cytometry experiments. In the case of a damaged cell membrane, the IncuCyte^®^ Cytotox dye enters the cell, intercalates into DNA, and thereby marks out the nuclei. Red fluorescence of the cytotoxicity dye can be measured at an excitation maximum of 612 nm and an emission maximum of 631 nm. As in flow cytometry experiments, apoptotic cells were analyzed by detection of active caspase-3/7. IncuCyte^®^ Caspase-3/7 reagent was used for the detection of active caspase-3/7, which is expressed and activated in apoptotic cells. Apoptotic cells can be identified by measuring green fluorescence at an excitation maximum of 500 nm and an emission maximum of 530 nm. The apoptosis inducer cisplatin was added in three wells to a final concentration of 50 µm to regular cell culture medium, in order to represent a positive control for apoptosis. MSCs cultivated in regular cell culture medium, without contact to 3D printing material, served as a control. As soon as the staining reagents with the corresponding medium were added to the cell culture wells, the monitoring was started using the IncuCyte^®^ S3 Live-Cell Analysis System. Phase contrast and fluorescence images were automatically captured every hour for a duration of 30 h. The experiment was performed with six biological replicates, every measurement in triplicates. Quantitative analyses of caspase-3/7 and cytotoxicity signals, as well as of cell proliferation were performed with pre-defined cell-specific masks in the IncuCyte^®^ S3 image analysis software.

## 3. Results

3D printed polyacrylic material was post-processed using three different sterilization or disinfection methods. To evaluate the efficiency of each post-processing and disinfection method as well as to investigate potential leaching properties of the 3D printed polyacrylic material itself, a comparative study using a biochemical-based standard plate reader assay (CTB Assay), standard flow cytometry, and an image-based live-cell analysis system was conducted. The leaching of acrylate monomers, degradation products, or other components from polymer-based materials is a well-known problem that often has negative effects on the biological environment [7,36,37,38]. Leachables can lead to cytotoxic effects on cells (which can manifest as irritations and/or allergic reactions within the human body) [8,36,39].

### 3.1. Biochemical-Based CTB Cell Viability Assay

**Figure 4:**
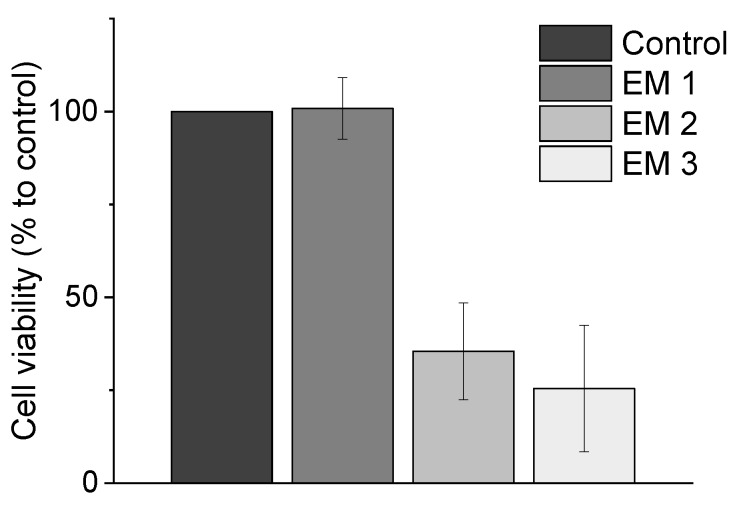
Results of CellTiter-Blue^®^ cell viability assay (CTB assay) to analyze the metabolic capacity (shown as cell viability in %) of MSCs. (EM = extraction medium). EM 1: EM obtained by incubation of 3D printed material treated with ethanol (70%, *v*/*v*) in a disinfection process. EM 2: EM obtained by incubation of 3D printed material treated with sodium hypochlorite (2%, *v*/*v*). EM 3: EM obtained by incubation of 3D printed material sterilized by UV light. All experiments were repeated several times (*n* = 13) and compared to MSC cultivation in regular cell culture medium (Control).

Metabolic activity as an indicator of cell viability of MSCs is analyzed by performing biochemical-based CTB cell viability assays during cultivation in extraction medium, which is prepared according to EN ISO 10993-12 (2012) (see Section 2.5). This CTB assay presents a biochemical-based method for assessing the cytotoxicity of a material. These results are summarized in Figure 4, where the cell viability observed during MSC cultivation in different extraction media is plotted. The cell viability is normalized to the control cultivation. Here, the use of ethanol (70%, *v*/*v*) (EM 1) as disinfectant did not show a significant difference in metabolic capacity and cell viability compared to control cultures. By contrast, both chemical disinfection methods of the 3D printed objects with sodium hypochlorite (2%, *v*/*v*) (EM 2), and irradiation sterilization (EM 3), caused a significant decrease in metabolic activity—resulting in only 35.5 ± 13,0% and 25.4 ± 17.0% viable cells, respectively, when compared to the control culture. From these results, the following conclusions could be drawn: (1) cleaning and disinfection of the 3D printed parts using ethanol 70% was successful, and (2) EM 1 did not contain any toxic leachables.

It can further be concluded that UV light is not a suitable sterilization method for the 3D printed material used in this study. The negative effects of EM 3 on cell viability may be due to several factors. UV light can have an adverse effect on both the optical and mechanical properties of polymer materials [40,41]. In our experiments, a slight change in color and translucency, as well as an increased brittleness of the surface of the material, was noticed after only 1 h of UV light exposure. Applications involving polymers are restricted due to the capability of photo-degradation, particularly under exposure of UV light [41,42]. Photooxidative reactions aroused by UV light are also associated with the formation of free radicals, which can lead to a radical chain mechanism and ultimately result in the rupture of a polymer structure. The degree of impact depends on the UV light intensity and duration—but this process initially manifests as a change in the color and an increased degree of “mistiness” observed in the polymer material [41,43]. These reactions may also lead to a release of leachables, which can have cytotoxic effects on cells. It should also be noted that the UV sterilization method was also rather impractical in this instance, because the 3D printed parts had to be rotated permanently in order to ensure uniform UV exposure. Since it would be difficult to maintain uniform UV irradiation across all surfaces of complex 3D printed structures—such as embedded channels in microfluidic systems—they would therefore be difficult to sterilize using this procedure.

Similarly, although sodium hypochlorite is the most widely used disinfectant in the food industry and a commonly used irritant in endodontic practice, a significant decrease in cell viability of MSCs was observed in our CTB assays after cultivation in EM 2 using sodium hypochlorite as a disinfection agent in the post-processing process [10,44,45,46]. This is perhaps not surprising; a study on mesenchymal stem cells of the human bone marrow from Alkahtani et al. has previously shown that even low concentrations of sodium hypochlorite exhibit cytotoxicity [47]. Treatment of sodium hypochlorite can thus damage cell membrane proteins and lead to cell lysis [48]. Such damage might have been responsible for the decreased metabolic activity observed in our MSCs. In contrast, the use of ethanol (70%, *v*/*v*) as a disinfection agent in the post-processing process of the 3D printed polyacrylic material has no negative impact on metabolic capacity of MSCs. Ethanol functioned as an effective disinfectant here without impacting either the optical or mechanical properties of the material. In addition, ethanol (70%, *v*/*v*) is also already a commonly used disinfectant in the health services field [23,49].

The CTB assay can score with its fast and user-friendly implementation while also allowing for high-throughput screenings. As a method performed in a standard plate reader, there is no need of sophisticated instruments. However, there is one important limitation on the CTB assay: it only provides information about the count of viable cells, and it is not sensitive to measuring the different mechanisms that can lead to cellular death, which present important information about the material formulation under investigation. Accordingly, to more precisely consider the impact of the post-processed 3D printed material on cell behavior, further studies aimed at measuring the rate of specific death mechanisms (i.e., apoptosis and necrosis) were also necessary. The use of specific dyes which mark out particular apoptotic and necrotic intracellular signals allowed for more detailed evaluations of cellular behavior and cytotoxicity mechanisms to assess in vitro biocompatibility. The standard plate reader used for CTB assays is not capable of detecting multiple fluorescence signals simultaneously. The follow section therefore considers the practicability of performing apoptosis and necrosis staining and analyses in a flow cytometry study vs. using a novel high-throughput image-based analysis system.

### 3.2. Analysis of Cell Death Responses via Flow Cytometry

**Figure 5:**
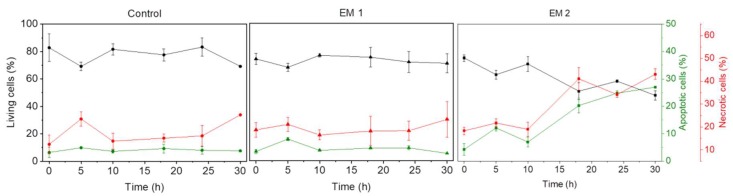
Results of flow cytometric studies on apoptosis and necrosis of MSCs over a period of 30 h. The percentage of living, apoptotic and necrotic cells are analyzed per cultivation. A caspase 3/7 signal (green) represents apoptotic cells; the cytotox-signal (red) is correlated to necrotic cells. (EM = extraction medium). EM 1: EM obtained by incubation of 3D printed material treated with ethanol (70%, *v*/*v*) in a disinfection process. EM 2: EM obtained by incubation of 3D printed material treated with sodium hypochlorite (2%, *v*/*v*). The experiments are compared to MSC cultivation in regular cell culture medium (Control) and were performed three times (*n* = 3).

Flow cytometry is a standard method used to monitor and quantitatively examine cell death via apoptosis and necrosis [29]. As cells undergoing necrosis experience a disruption of the cell membrane, the use of a red fluorescence dye that enters and labels the DNA of damaged cells with disrupted cell membranes is an elegant and effective way to visibly mark out such cells [33]. Specific fluorescence labeling can also be used to visually detect apoptotic cells, which express and activate the enzymes caspase-3 and caspase-7 [33]. Here, a green fluorescence dye that is sensitive to active caspase-3/7 was used to identify apoptosis (see Section 2.8.2). The relative percentage of necrotic vs. apoptotic MSCs within a sample can then be assessed and used to analyze the biocompatibility of the 3D printed material after post-processing and disinfection (see Section 2.5). Figure 5 shows the flow cytometric analysis of MSCs cultivated over a period of 30 h.

As explained above (see Section 3.1), UV light is not a suitable sterilization method for the 3D printed material used in this study. Therefore, as shown in Figure 5, UV light as sterilization method was no longer analyzed. MSCs that were cultivated in extraction medium 1 (EM 1), obtained by incubation of 3D printed material disinfected by ethanol (2%, *v*/*v*), showed no significant difference with respect to the relative percentages of living, apoptotic, and necrotic cells when compared to control cultures; in both cases, the percentage of apoptotic cells was about 4%, the percentage of necrotic cells was about 16%, and the balance were living cells. Since the same number of cells was stained and used for each measurement, the data does not show any increase in the count of living cells due to cell growth. In contrast to the MSCs in EM 1 and the control cultures, the cultivation of MSCs in extraction medium 2 (EM 2)—obtained by incubation of 3D printed material disinfected by sodium hypochlorite (2%, *v*/*v*)—resulted in a strong increase in both apoptotic and necrotic cells. In this medium, the percentage of apoptotic and necrotic cells increased over time from 4% and 18%, respectively, to approximately 30% and 45%, while the percentage of living cells correspondingly decreased from 80% to 50%. Each experiment showed a slight increase in the percentage of apoptotic and necrotic cells, as well as a simultaneous decrease in the count of living cells (after 5 h). This occurrence may be related to the change of cell culture medium to relevant extraction or control medium, and adaption of the cells to their new environment—which is associated with cellular stress [50].

**Figure 6:**
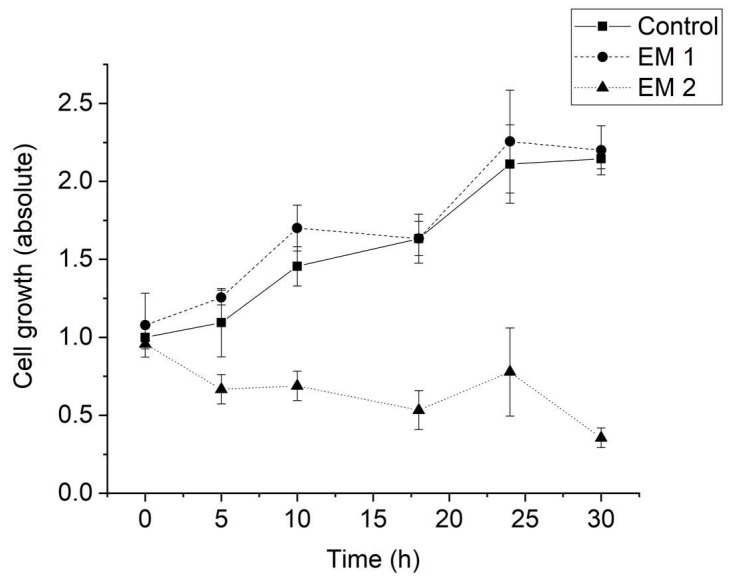
Cell growth of MSCs determined by cell counting using Trypan blue staining. (EM = extraction medium). EM 1: EM obtained by incubation of 3D printed material treated with ethanol (70%, *v*/*v*) in a disinfection process. EM 2: EM obtained by incubation of 3D printed material treated with sodium hypochlorite (2%, *v*/*v*). The experiments are compared to MSC cultivation in regular cell culture medium (Control) and were performed three times (*n* = 3).

Figure 6 illustrates the calculated cell growth over a cultivation period of 30 h. For MSCs cultivated in EM 1, no significant difference in cell growth was observed when compared to control cultures. Over the cultivation period, the number of living cells increased by a factor of approximately 2, both for cultivation in control medium and in EM 1. By contrast, cultivation in EM 2 leads to a strong decrease in cell viability, which resulted in a significant decrease in the number of living cells (by more than half) within 30 h.

In summary, then, apoptosis/necrosis analyses over 30 h reveal no evidence of any behavior in MSCs cultivated in EM 1 that could be attributed to potential toxic leachables in the 3D printed material. And a post-processing procedure that included disinfection with ethanol (70%, *v*/*v*) proved to be the most advisable approach tested for handling this high-resolution polyacrylic 3D printed material. In general, the flow cytometry results confirm the results of the CTB assay, but it provides more detailed information about the mechanism of cell death that was observed.

### 3.3. Analysis of Cell Death Responses via Image-Based Live-Cell Analysis System

Another approach for analyzing apoptotic and necrotic responses of cells in order to assess in vitro biocompatibility of a material is represented by comparatively novel image-based live-cell analysis systems. The IncuCyte^®^ Live-Cell Analysis System used in this study is an image-based real-time system that allows the automatic acquisition and analysis of phase contrast and fluorescence images of cells using customized software tools.

**Figure 7:**
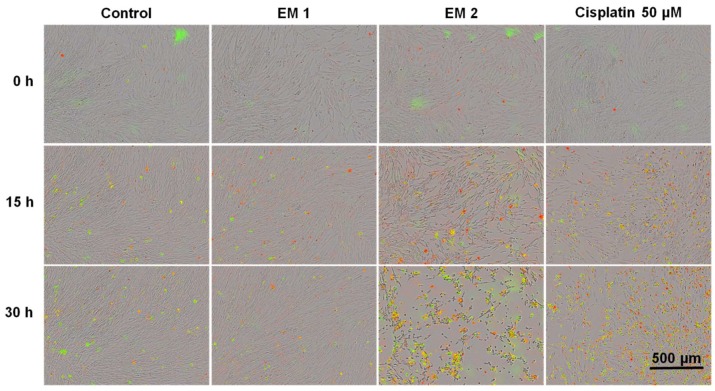
Fluorescence images of MSCs over time by image-based live-cell analysis system (IncuCyte). Green fluorescence is related to apoptotic cells; red fluorescence shows necrotic cells. (EM = extraction medium). EM 1: EM obtained by incubation of 3D printed material treated with ethanol (70%, *v*/*v*) in a disinfection process. EM 2: EM obtained by incubation of 3D printed material treated with sodium hypochlorite (2%, *v*/*v*). The experiments are compared to MSC cultivation in regular cell culture medium (Control) and were performed three times (*n* = 3). Cisplatin 50 µm: Positive control for apoptosis.

Using this system, MSCs cultivated either in extraction or in a control medium (see Section 2.5) were monitored and analyzed automatically over a period of 30 h. Phase contrast, as well as fluorescence images, were captured every 1 h following the addition of fluorescence reagents for the purpose of highlighting apoptosis and necrosis. A contrasting juxtaposition—representing the cell phenotype data of individual cell populations cultivated in extraction or control medium—is shown in Figure 7 Green fluorescence signals show apoptotic cells; red fluorescence signals show necrotic cells. As a positive apoptosis control, MSCs were cultivated with the addition of the apoptosis inducer cisplatin. In keeping with previous investigations (see Section 3.1 and Section 3.2 above), no differences in cell morphology, cell growth, or layer formation was observed for MSCs cultivated in EM 1 compared with control cultures. By contrast, MSCs cultivated in EM 2 show similar characteristics compared to the cultivation of MSCs with cisplatin (positive apoptosis control). After 15 h of incubation in EM 2 or cisplatin, large gaps in cell layer, less connected cells, and cell rounding as well as shrinkage were all observed. These are common characteristics associated with cell apoptosis [51]. After 30 h of MSC cultivation in EM 2 and the positive apoptosis control, a high increase in apoptotic and necrotic signals was observed via measurements of corresponding fluorescence signals.

**Figure 8:**
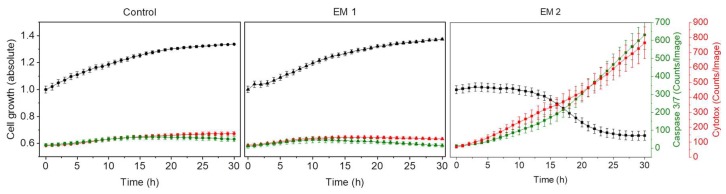
Analysis of cytotoxicity of the 3D printing polyacrylic material and apoptosis of MSCs by an image-based live-cell analysis system (IncuCyte). Cell growth, apoptosis and necrosis of MSCs are analyzed per cultivation. A caspase 3/7 signal (green) represents apoptotic cells; the cytotox-signal (red) is correlated to necrotic cells. (EM = extraction medium). EM 1: EM obtained by incubation of 3D printed material treated with ethanol (70%, *v*/*v*) in a disinfection process. EM 2: EM obtained by incubation of 3D printed material treated with sodium hypochlorite (2%, *v*/*v*). The experiments are compared to MSC cultivation in regular cell culture medium (Control) and were performed eighteen times (*n* = 18).

Figure 8 shows kinetic analyses of MSC growth, as well as apoptotic and necrotic signals obtained by dynamic image processing of phase contrast and fluorescence images, as described in Section 2.9. In this Figure, the unit Counts/Image was based on fluorescence signals provoked by apoptotic or necrotic cells in a specific imaging field. MSCs were cultivated in corresponding extraction media or control medium (see Section 2.5, above). As was to be expected from the previous investigations, there was no relevant difference observed in the cell behavior of MSCs cultured with EM 1 compared to the control cell culture medium. Over the duration of the experiment, cell confluency (representing the cell growth) increased. Living cells grow, expand, and divide. Furthermore, the number of apoptotic and necrotic cells per image field during MSC cultivation in EM 1 and control medium remained minimal. By contrast, MSC cultivation in EM 2 stagnated, and a strong relative increase in apoptotic and cytotoxic signals was also observed. A subsequent decline in cell proliferation after 10 h in EM 2 was likely related to the changes in cell morphology (e.g., cell rounding, shrinkage) and detachment of dead cells from the surface as a result of increased apoptosis and necrosis [14]. Detached dead cells might migrate into the supernatant, beyond the focal point of the laser, where they cannot be recognized and counted adequately.

## 4. Discussion

The results obtained from the image-based evaluation conducted via live-cell analysis system were in full agreement with the results obtained via both the CTB assay and flow cytometry method—and all three methods confirmed that EM 1 had no significant influence on MSCs. It can therefore be assumed that the post-processing procedure including disinfection with ethanol (70%, *v*/*v*) was successful, and no critical amount of cytotoxic substances leached out of the 3D printed polyacrylic material. Since the 3D printed polyacrylic material had no negative impact on cell behavior or cell morphology of MSCs, it can be considered in vitro biocompatible. These findings collectively mark out a solid starting point for further investigations, and open the door for potential biological and biomedical applications using the analyzed 3D printed high-resolution polyacrylic material, which is promising not only for micro-scale and microfluidic applications, but also for rapid prototyping of various devices for cell culture and lab scale experiments [17].

Comparing the three methodologies used to evaluate in vitro biocompatibility here reveals some major disadvantages of both the flow cytometry and CTB assay methods. In both oh those cases, cell sample preparation and analysis must take place outside the cell culture incubator, which is designed to ensure a constant temperature and high humidity to facilitate cell growth under a CO_2_ atmosphere. Such handling of the cells outside the incubator disrupts these optimal conditions, which may impose cellular stress and could also potentially impact cell growth, apoptosis, and/or necrosis [52,53]. Furthermore, as noted above, analysis of biocompatibility via biochemical-based CTB assay only provides information about cell viability in general. As a result, it can at best be considered a first analysis assay useful to obtaining a general sense of the cytotoxicity potential of a material, before continuing with further considerations. Flow cytometry and the image-based analysis system both allow for more detailed and specific analyses of cellular behavior and reactions on potential cytotoxic material constituents in assessing biocompatibility. For flow cytometry experiments, the cells of an individual cultivation well were harvested and examined for each measuring point. This means that in flow cytometry analyses, different cell populations are compared with each other, and therefore temporal investigation based only on a single cell population is not possible (this applies to adherent growing cells). Attempting to track dynamic functional cellular processes and morphology over the whole time frame of an experiment accordingly becomes an arduous task; and flow cytometry is particularly ill-suited to the task of monitoring rapid cellular changes (e.g., in response to external influences). Due to well-to-well variations and differences in cell treatment and seeding, the comparability of the obtained data cannot be guaranteed [54]. In addition, sample preparation for flow cytometry studies is laborious, requiring substantial time expenditure and good cell culture practices [54]. The extensive sample handling also results in a substantial delay from cell detachment to analyses. Additionally, the multiple centrifugation steps required during sample preparation and dyeing procedures expose the cells to mechanical stress [54]. Disruption and damage of the cell membrane triggered by stress factors can lead to apoptotic or necrotic responses and thus to false-positive results.

In contrast, image-based live-cell analysis gives the ability to visualize cellular phenotypes images as well as to perform kinetic analyses and quantifications of apoptotic and necrotic cell responses simultaneously in high-throughput. Based on microscopic data, numerous cell specific analyses can be performed directly, using customized tools and software. Live-cell imaging technology offers the possibility to monitor and study the same cell population for an indefinite period of time by analyzing the same imaging field. Since the imaging and analysis is realized fully automated inside a cell culture incubator, there is no need to physically move cells and risk exposing them to lower temperatures and potential cellular stress. Culture perturbations in performing assays with traditional methods such as flow cytometry and CTB viability assays are affecting cellular behavior and provoke cellular stress [52,53,54]. That includes the physical movement of cultures by removing cell culture flasks or plates from the laboratory cell culture incubator as well as changes in temperature and atmospheric conditions while performing the experiment. The real-time analysis system does not have to take into account any of the aforementioned disturbances.

## 5. Conclusions

This study presents a comprehensive comparison of three different methodologies for the in vitro evaluation of biocompatibility of 3D printed polyacrylic material. The superiority of an image-based live-cell analysis system with respect to time, usability, and scientific outcome was shown. Image-based real-time analyses allow for simultaneous observations of changes in cell morphology via microscopic imaging as well as kinetic analyses and quantifications of apoptotic and necrotic cell responses. Conventional methods for testing in vitro biocompatibility—such as microscopy or biochemically based assays—were comparatively outshone. The fast and simple handling; the potential of performing screenings in high throughput; and the high quantity and informative value of cellular data all make real-time live-cell imaging technology an ideal tool not only for the study of biocompatibility, but also for the usage in numerous cell culture applications on a daily basis. With the possibility of integrating up to two fluorescence channels in addition to phase contrast, and the choice between three different objective nosepieces, countless image-based cell assays can potentially be performed and monitored in real-time. Long term assays for studying chemotaxis, angiogenesis or stem cell differentiation are just as simple to realize as measurements of cellular health in drug screenings.

At the same time, this study also highlighted the importance of analyzing and comparing different post-processing procedures of 3D printed materials considered for biological applications Even though the tested material itself is in vitro biocompatible, remaining support material or contaminations due to insufficient post-processing methods could still potentially lead to adverse effects on surrounding cellular environment. 3D printing materials produced for a specific printer system are often not considered for use in cell culture or biomedical applications where biocompatibility is a central demand [3]. Manufacturers often give no suggestions for a proper disinfection and sterilization of their numerous material formulations. It is accordingly up to the researcher to investigate the materials in terms of biocompatibility and appropriate post-processing and sterilization protocols. On that account, high-throughput screening methods as the image-based live-cell analysis system are critical for both finding biocompatible material formulations, and also finding the best solution of post-processing for one given material.
